# The Geographic Synchrony of Seasonal Influenza: A Waves across Canada and the United States

**DOI:** 10.1371/journal.pone.0021471

**Published:** 2011-06-28

**Authors:** Dena L. Schanzer, Joanne M. Langley, Trevor Dummer, Samina Aziz

**Affiliations:** 1 Infectious Disease Prevention and Control Branch, Public Health Agency of Canada, Ottawa, Ontario, Canada; 2 Canadian Centre for Vaccinology, IWK Health Centre and Faculty of Medicine, Dalhousie University, Halifax, Nova Scotia, Canada; 3 Population Cancer Research Program, Department of Pediatrics, Dalhousie University, Halifax, Nova Scotia, Canada; Duke-National University of Singapore, Singapore

## Abstract

**Background:**

As observed during the 2009 pandemic, a novel influenza virus can spread globally before the epidemic peaks locally. As consistencies in the relative timing and direction of spread could form the basis for an early alert system, the objectives of this study were to use the case-based reporting system for laboratory confirmed influenza from the Canadian FluWatch surveillance program to identify the geographic scale at which spatial synchrony exists and then to describe the geographic patterns of influenza A virus across Canada and in relationship to activity in the United States (US).

**Methodology/Principal Findings:**

Weekly laboratory confirmations for influenza A were obtained from the Canadian FluWatch and the US FluView surveillance programs from 1997/98 to 2006/07. For the six seasons where at least 80% of the specimens were antigenically similar, we identified the epidemic midpoint of the local/regional/provincial epidemics and analyzed trends in the direction of spread. In three out of the six seasons, the epidemic appeared first in Canada. Regional epidemics were more closely synchronized across the US (3–5 weeks) compared to Canada (5–13 weeks), with a slight gradient in timing from the southwest regions in the US to northeast regions of Canada and the US. Cities, as well as rural areas within provinces, usually peaked within a couple of weeks of each other. The anticipated delay in peak activity between large cities and rural areas was not observed. In some mixed influenza A seasons, lack of synchronization sub-provincially was evident.

**Conclusions/Significance:**

As mixing between regions appears to be too weak to force a consistency in the direction and timing of spread, local laboratory-based surveillance is needed to accurately assess the level of influenza activity in the community. In comparison, mixing between urban communities and adjacent rural areas, and between some communities, may be sufficient to force synchronization.

## Introduction

Influenza epidemics occur annually with peak activity occurring from November to April in temperate climates of the northern hemisphere, affecting 5–20% of the population [1]–[4] and causing considerable morbidity [5], [6] and mortality [7]. Influenza A viruses contribute to most of the disease burden, although influenza B is also associated with medically attended illness [8]. In some seasons, a single antigenic strain is responsible for the annual epidemic and in others, multiple strains account for a significant proportion of confirmed cases.

Recent studies have identified a strong synchrony in influenza activity between adjacent jurisdictions using a variety of indicators for influenza activity such as peak pneumonia and influenza admissions [9], peak pneumonia and influenza deaths [10], or peak clinical activity (consultations for influenza like illness (ILI)) [11]. As regional data points can be sparse, spatial interpolation methods have been used to facilitate mapping. These maps, perhaps as a result of the interpolation, indicate a smooth geographic spread of disease outward from a point source [12]. Many jurisdictions, including Canada [8], the United States [13] and Europe [14] provide near real time maps of the geographic spread of influenza activity by influenza surveillance regions through web portals. These maps can be based on a variety of indicators of influenza activity such as excess ILI consultations, institutional outbreaks, or laboratory confirmation of cases. Activity is considered “widespread” when influenza activity is observed in one or more sub-region(s) accounting for at least 50% of the population of the region [13]. In contrast to the maps produced using spatial interpretation, these maps will often indicate sporadic or localized activity within a surveillance region. “Localized” activity suggests that within a surveillance region, some communities are experiencing influenza activity and others are not, that is, geographic spread is highly irregular. Depicting yet another description of geographic spread, Bonabeau and colleagues [15] found evidence suggestive of a synchronized epidemic for most of the country based on influenza case reports from a large network of general practitioners in France.

We used the case-based laboratory data from the Canadian *FluWatch* influenza surveillance program from which the community of residence of cases testing positive for influenza was available [16] to identify the geographic scale at which spatial synchrony likely exists. To describe the patterns of spread across larger geographic regions, we used case counts available at the provincial level in Canada and for the nine influenza surveillance regions covering the United States.

Various hypotheses have been put forward to explain the seasonality and transmission patterns of influenza [17]. Rapid diffusion of influenza over long distances has been attributed to the global transportation network, and modelling the geographical spread based on global [18], [19] and local [20] transportation networks produces predictable patterns of spread. Since such models predict that epidemics would start in the large urban centres that have the strongest international connections to the source country or countries, we aimed as well to explore the level of synchrony between communities of different sizes.

## Methods

### Sources of data

Canadian data was obtained from two related surveillance systems that are part of the *FluWatch* program of the Public Health Agency of Canada [8], [16]. Weekly counts of the number of influenza positive test results by province were obtained from the Respiratory Virus Detection Surveillance System (RVDSS), which collects laboratory test results from participating laboratories across the country. Most participating laboratories also provide additional non-identifying epidemiological information on individual cases through the case-based reporting system [16]. The specimens are submitted to laboratories for viral identification by clinicians in the course of clinical care in inpatient, emergency room or outpatient settings, and by sentinel physicians participating in the national influenza surveillance program, *FluWatch* [8]. Community names corresponding to the community of residence from the case-based reporting system were grouped into census metropolitan areas (CMA), census agglomerations (CA), and rural areas using a standard geographic coding system [21]. A CMA/CA is an area consisting of one or more neighbouring municipalities situated around a major urban core with an urban population of at least 10,000 for a CA and 100,000 for a CMA.

In the United States, specimens are tested for influenza in U.S. World Health Organization (WHO) Collaborating Laboratories and in the laboratories of the National Respiratory and Enteric Virus Surveillance System [13]. The results were posted weekly by the Center for Disease Control (CDC) for each of 9 surveillance regions (Pacific, Mountain, West South Central, East South Central, West North Central, East North Central, New England, Mid Atlantic, and South Atlantic) as part of the FluView weekly influenza surveillance report.

### Analysis

Analysis was limited to seasons where a single antigenic strain accounted for at least 80% of the influenza A strains characterized, in order to avoid the potential situation of a bi-modal epidemic curve that could result from the co-circulation of two different influenza A strains (for example an H1N1 strain and an H3N2 strain). Data for influenza B epidemics was not included in the analysis, as influenza B accounted for only a small proportion of all influenza positive tests.

The midpoint of the epidemic, defined as the week when the cumulative incidence reaches 50% of the cumulative seasonal total, was chosen as the reference point for the timing of the regional or local epidemic for the following reasons. Firstly, differences in detection rates across communities would not bias the epidemic midpoint statistic calculated for each community. Secondly, as a statistical measure, the median (epidemic midpoint) is less volatile than the maximum (week with the most number of cases), of particular concern for communities with a small number of confirmed cases. Though the association between the epidemic midpoint corresponding to a particular community for a single season and the natural epidemic peak is not observable, a simulation using the empirical epidemic curve estimated in a previous Canadian study [22] suggests that the observed epidemic midpoint in a community with only 50 confirmed cases would correctly identified the natural epidemic peak of the underlying population to within one week 83% of the time.

### Regional Patterns

The epidemic midpoint was determined for the following geographic units: Canadian province, and US surveillance region. The geographic unit or “region” that peaked first in each season was identified as the reference region, and the delay in weeks by region was plotted on a choropleth map of Canada and the United States. Choropleth maps are maps that use colour, shading or patterning to display specific statistics for defined areal units, such as Canadian Provinces and US Surveillance Regions. In this case, four statistics of interest were displayed using chloropleth maps: the delay in timing of the epidemic compared to the lead region or reference region (measured by the delay in weeks of the epidemic midpoint) by influenza season; the average calendar week of the epidemic midpoint over the study period; and the minimum and maximum calendar weeks to show the full range of variability from season to season. Calendar weeks were numbered according to the calendar year with week 1 corresponding to the first week of January. Weeks run from Sunday to Saturday and run continuously so that each week covers a 7 day period. The influenza season starts with week 35 or the 1st week of September and runs for a full year. To calculate the average calendar week, weeks were first renumbered sequentially over the season and the average converted back to calendar week.

### Sub-provincial Patterns in Canada

Sites (CMA/CA) reporting at least 50 influenza A confirmations per season were included in the community level analysis as separate geographic units for that season. Because of this inclusion criterion, the number of geographic units varied from season to season. Cases from communities not meeting the inclusion criteria in any one season were grouped together at the provincial level into one of three geographic units: residual CMA, residual CA, and rural areas. The number of confirmed cases per community was not considered geographically representative, as influenza testing procedures, reporting practices, and the availability of diagnostic services varied by jurisdiction. The epidemic mid-point was determined for each Canadian city (CMA/CA) or rural or residual urban area with at least 50 influenza positive cases during the season. Synchronization at the community level was assessed by calculating the difference in timing of the epidemic midpoint between the largest CMA and all other communities within the province. The list of local epidemics that were out of synchronization with the largest CMA in the province by more than 2 weeks was reviewed for any potential patterns. For a formal test of association, the Chi-squared test of association was used to test for the effect of community type on synchronization.

The choropleth maps were produced using the ArcView 9.2 GIS (geographic information system) mapping software system (ESRI, Redlands, CA). Statistical computations were carried out using SAS Enterprise Guide 4.1 (The SAS Institute, Cary, NC).

## Results

The number of influenza A positive test reports varied 10-fold from season to season over the 10 seasons (1997/98 to 2006/07), averaging approximately 6,000 positive test reports per season in Canada and 12,000 in the United States.

### Regional Patterns

**Figure 1:**
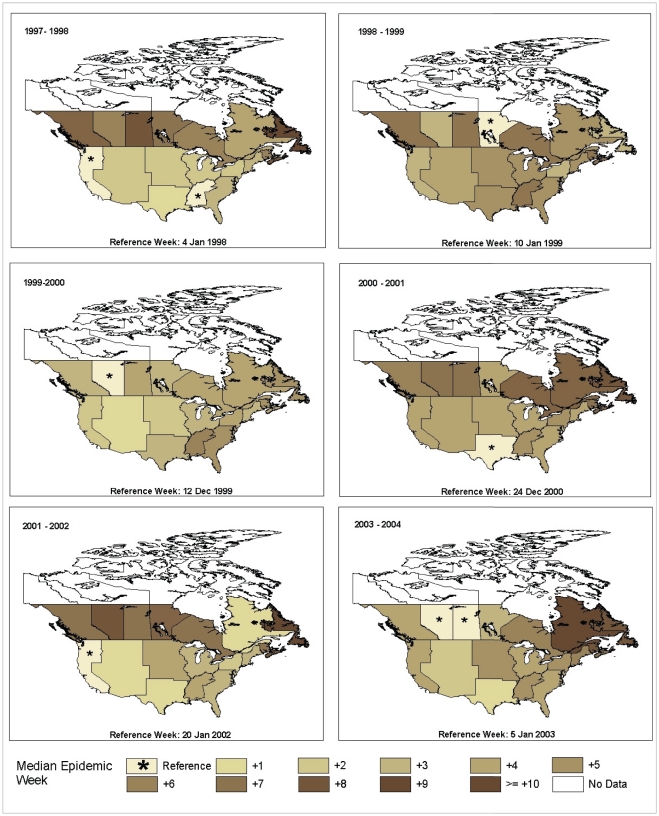
Timing of the Annual Influenza A Epidemic across Canada and the United States, by Season. The temporal midpoint of each influenza A season where over 80% of the influenza strains were antigenically similar by Canadian province and American influenza surveillance region is shown in the chloropleth maps. The region reaching its midpoint first was identified as the reference region. In three out of the six seasons the epidemic appeared first in Canada. Considerable variability in the timing and direction of spread is noted.

From national year-end summary reports, the following six seasons met the dominance criteria (a single antigenic strain accounted for at least 80% of the influenza A strains characterized) in both Canada and the United States: 1997/98, 1998/99, 1999/2000, 2000/01, 2001/02, and 2003/04 [8], [13], [23]–[25]. With the exception of two mixed A seasons (2004/05 and 2006/07), the predominant influenza A strains circulating in Canada and the US were similar (Table 1). The timing and spread of the annual influenza epidemic across Canada and the continental United States for these six seasons is shown in the chloropleth maps (Figure 1). As seen from the different reference regions over time, the geographic region that experienced the earliest epidemic wave varied over the six influenza seasons. In three out of the six seasons the epidemic appeared first in Canada (Table 2); in the other three seasons, the peak occurred first in the US. Across US regions, the difference in timing of the epidemic midpoint ranged from 3 to 5 weeks. In comparison, provincial differences in timing across Canadian provinces ranged from 5 weeks in the 1999/00 A/Sydney/5/97 H3N2 season and the 2000/01 A/New Caledonia/20/99 H1N1 season to 12 weeks in the 2003/04 A/Fujian/411/02 H3N2 season.

**Table 1:** Strain type and composition.

	Canada			United States		
Season	Predominant A strain	Sub-Type	% of Influenza A specimens	Predominant A strain	Sub-Type	% of Influenza A specimens
**1997/98**	A/Sydney/05/97	H3N2	82%	A/Sydney/05/97	H3N2	81%
**1998/99**	A/Sydney/05/97	H3N2	99%	A/Sydney/05/97	H3N2	90%
**1999/00**	A/Sydney/05/97	H3N2	83%	A/Sydney/05/97	H3N2	84%
**2000/01**	A/New Caledonia/20/99	H1N1	97%	A/New Caledonia/20/99	H1N1	85%
**2001/02**	A/Panama/2007/99	H3N2	82%	A/Panama/2007/99	H3N2	93%
2002/03	A/New Caledonia/20/99	H1N1	79%	A/New Caledonia/20/99	H1N1	67%
**2003/04**	A/Fujian/411/02	H3N2	99.5%	A/Fujian/411/2002	H3N2	89%
2004/05	A/Fujian/411/02	H3N2	56%	A/California/7/2004	H3N2	77%
2005/06	A/California/7/2004	H3N2	72%	A/California/7/2004	H3N2	59%
2006/07	A/Wisconsin/67/2005	H3N2	59%	A/New Caledonia/20/99	H1N1	57%

Seasons where over 80% influenza A strains were antigenically similar are shown in **BOLD**.

**Table 2:** Reference Region: Region with the Earliest Epidemic.

				Earliest Epidemic Midpoint		Minimum delay(in weeks)		Difference in Synchronization(in weeks)	
Influenza Season	Type	Country	Region	Date	Week	To Canada	To US	Across US	Across Canada
1997/98	H3N2	US	Pacific, Eastern South Central	4-Jan-98	1998-02	5		3	6
1998/99	H3N2	Canada	Manitoba	10-Jan-99	1999-02		3	3	7
1999/00	H3N2	Canada	Alberta	12-Dec-99	1999-50		1	5	5
2000/01	H1N1	US	West South Central	24-Dec-00	2000-52	4		5	5
2001/02	H2N2	US	Pacific	20-Jan-02	2002-04	1		4	10
2003/04	H3N2	Canada	Alberta, Saskatchewan	9-Nov-03	2003-46		4	5	12

**Figure 2:**
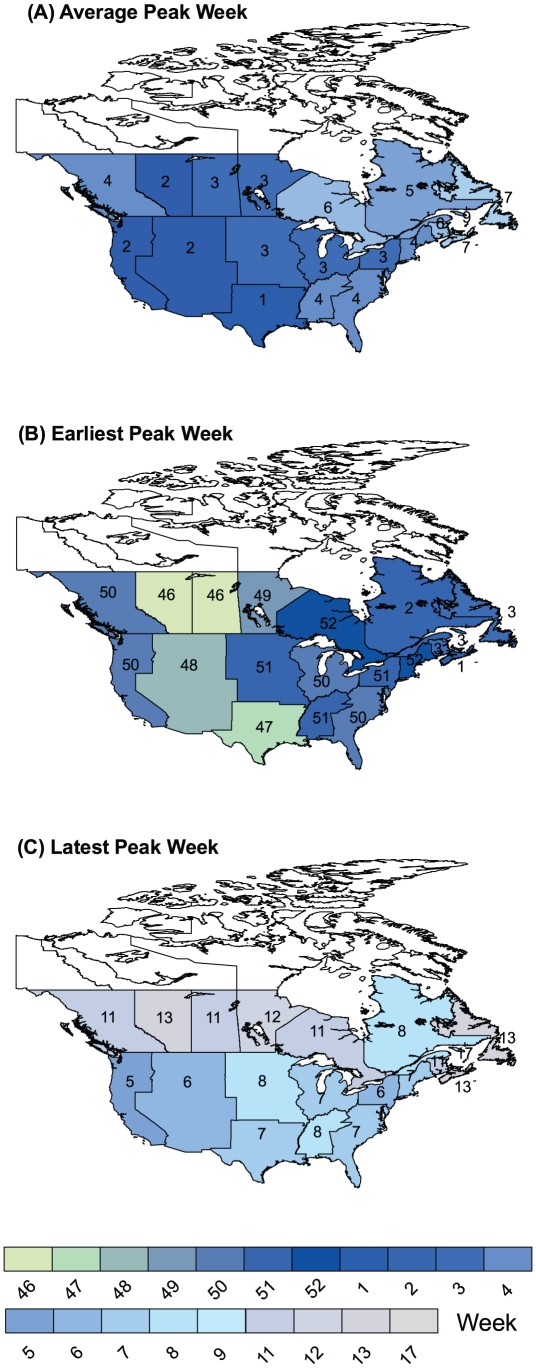
Summary of the Timing of the Annual Influenza A Epidemic across Canada and the United States. Weeks are numbered according to the calendar year with week 1 corresponding to the 1^st^ week of January and week 52 is one week earlier. Weeks run from Sunday to Saturday, and run continuously so that each week covers a 7 day period. The influenza season starts at week 35 or the 1st week of September and runs for a full year. Week 53 was not included in the legend as it only occurred in 2 out of 6 seasons. Only seasons where over 80% of the influenza strains characterized were antigenically similar are included in the summaries, namely, seasons shown in Figure 1: 1997/98, 1998/99, 1999/2000, 2000/01, 2001/02, and 2003/04. a) The average week of peak activity; b) The earliest week of peak influenza activity by Canadian province and American influenza surveillance region; c) The latest week of peak influenza A activity by Canadian province and American influenza surveillance region.

The average week of peak activity over the 6 seasons varied from week 1 (1^st^ week in January) in the West South Central region of the United States (Texas, Louisiana, Arkansas, and Oklahoma) to week 7 (mid February) in the eastern most provinces of Nova Scotia and Newfoundland and the chloropleth map for Canada and the US suggests a slight gradient in timing from the southwest to northeast (Figure 2a).

For individual seasons, there was considerable variation in timing, with epidemic waves peaking as early as week 46 (early November) in Canada and week 47 in the United States to as late as week 13 (end of March) in Canada and week 8 (mid February) in the United States (Figures 2b, c). Peak activity was not observed in the eastern Canadian provinces prior to the first week of January.

While epidemics in neighbouring regions were often closely synchronized to within a week or two, longer delays were also possible (Figure 1). On average the difference in timing between adjacent regions was only a couple of weeks, however, most seasons would see differences of 6 to 8 weeks between a few adjacent regions. In the US, the largest delay of 5 weeks was between the East South Central and West South Central regions. Longer delays of up to 8 weeks were seen between the Pacific or Mountain regions and the Canadian provinces to the north.

### Sub-provincial Patterns in Canada

The availability of the case-based dataset in addition to the summary data from provinces permitted a sub-provincial analysis of the Canadian data. Sub-regional data was not available for the United States. The case-based laboratory reporting system captured the city of residence for an average of 4,000 cases per season. Representation by community size was in reasonable agreement with the 2001 Canadian census [21]. CMAs accounted for 57% of the cases with epidemiological information, compared to 64% of the Canadian population. Rural areas were slightly over represented, with 28% of the case reports compared to 21% of the population. Most of the rural population lived in communities that were considered to be influenced by a neighbouring CMA/CA [26], [27]; over half of the rural population lived in communities where at least 5% of the labour force commuted to work in a nearby CMA/CA, and only 5% with less than 1% commuting to work in a nearby CMA/CA [26]. The number of CMA/CAs reporting more than 50 laboratory confirmed influenza A cases in a season varied: with 18 out of 33 CMAs and 4 out of 111 CAs reaching this threshold in the 2003/04 season, and only the two largest Canadian cities, Toronto and Montreal, in the mixed 2002/03 season. In the 6 seasons included in subsequent analyses, the number of CMA/CAs meeting the above criterion ranged from 12 to 22 CMA/CAs per season, for an average of 17. The threshold of 50 cases per community per season resulted in the exclusion of approximately 20% of the urban cases. For the few provinces with sufficient tests, the synchronization of cases from the excluded CMA, excluded CA, and rural regions were compared and these combined regions were found to be closely synchronized. Cases from these three residual categories were pooled, with the resulting residual category consisting primarily of cases from rural communities (57%). The number of influenza cases reported per season in some of the smaller provinces was limited. Even after pooling, only about half of the provinces had sufficient cases to compare the synchronization of the major urban centre with the rest of the province each season (32 ‘rest of province’ comparisons out of 10 provinces over 6 seasons, Table 3).

**Table 3:** Degree of Synchronization^1^ between the Major City^2^ in each Province and other Communities.^3^

	Number of community-level epidemics (≥50 cases)							
Synchronization Criteria	Rural Area/Rest of Province 4	(%)	CMAs, pop >100,000	(%)	CAs, pop 10,000–100,000	(%)	Total	(%)
within 1 week	17	53%	30	64%	17	49%	64	56%
within 2 weeks	26	81%	40	85%	29	83%	95	83%
within 3 weeks	30	94%	47	100%	33	94%	110	96%
Total Number of Epidemics Compared^5^	32	100%	47	100%	35	100%	114	100%

1 Includes seasons where over 80% of the influenza strains were antigenically similar.

2 The major city for each province was identified as the CMA reporting the most cases.

3 Communities (geographic units) which confirmed at least 50 positive cases in one season.

4 Cases from the rural area of each province and communities with less than 50 positive cases per season were combined into one local epidemic curve. Cases from rural areas accounted for the majority of cases in the ‘rest of the province’ epidemic curve.

5 The number of local epidemics meeting the inclusion criteria over the study period of 6 seasons. To compare sub-provincial synchronization at least 2 geographic units from one province must meet the inclusion criteria for the same season; that is at least 1 CMA/CA and one other community from the same province or the ‘rest of province’ aggregate must meet the criteria in the same season. Over the 6 seasons, 114 pairs were available to assess the degree of synchronization.

The Chi-squared value for a test of association is 2.3 on 4 degrees of freedom for a p-value of 0.67. Note that cell counts are given in cumulative format in this table and the categories of 3 weeks and 4 or more weeks were combined in the test of association due to small numbers.

The average difference in the timing between the epidemic waves of the largest city and other communities within the same province was usually a week or less (Table 3), and the local epidemics were synchronized to within less than 14 days in more than 80% of the communities. This degree of variation is slightly larger than would be expected by chance alone, but unlikely to be detectable using methods available for near real time reporting. City size was not associated with the timing of peak activity, as CMAs, CAs and smaller towns within a province peaked on average at the same time (Chi-squared test of association was not statistical significant, Table 3). No preference for direction of spread (not shown) was seen. The mixed season of 2004/05 was a notable exception to the close synchronization within provinces (results not shown).

## Discussion

This analysis shows the importance of considering geographic scale in the interpretation of our influenza surveillance data. Based on laboratory confirmations reported at the provincial/regional level, a slight gradient of spread across Canada and the US from the southwest towards the northeast was identified. However, the epidemic peaked in at least one Canadian province before peaking in an American region in 3 out of 6 seasons analysed, and the season-to-season variation in both the timing and direction of spread was more notable than the trends. While epidemic waves in adjacent regions tended to be closely synchronized, the degree of social mixing between adjacent regions appears to be too weak to force a consistency in the degree of synchronization between most pairs of adjacent regions. In comparison, mixing between urban communities and adjacent rural areas, and between some communities, may be sufficient to force synchronization. While influenza epidemic waves are generally closely synchronized for the major population areas within provinces, some sub-provincial differences were noted, particularly in seasons where more than one influenza A strain circulated. Currently available data is still insufficient to fully identify the geographic areas for which mixing is sufficient to ensure near synchronization. From commuter flow data from the 2006 census [26], only 10% of the Canadian population lives in a rural area where less than 5% of the labour force commutes to work in a nearby urban area. Theoretically, this degree of mixing between urban and rural areas could be sufficient to force near synchronization, though continued surveillance at a fine geographic scale is still needed to identify the appropriate geographic boundaries for synchronous regions.

Our results are in general agreement with studies that have assessed the spatial and temporal spread at the population level. Synchronization over large geographic regions has been previously noted in the US [9], [10], with a French study noting as well that diffusion appears to occur over long distances before the epidemic builds up within communities [15]. The relatively slower rate of spread across Canada is consistent with results for Europe [11]. A strong gradient in the timing of the local influenza epidemics has been noted within Brazil and globally [28], [29], where tropical and semi-tropical regions peaked earlier than adjacent temperate regions. A weak south-to-north spread was noted across Europe in some seasons only [11] and a similar southwest to northeast spread across continental US has been reported [9]. The long delays of up to 8 weeks seen between the Pacific or Mountain regions and the Canadian provinces to the north observed in this study may be due to variation in the timing of community level epidemics within these two American regions which stretch from the Mexican to Canadian border. Variation within these regions is supported by Wenger's and Naumova's [9] state level analysis of peak pneumonia and influenza admissions, and as of the 2008/09 season, states were regrouped into 10 surveillance regions.

The hypothesis that influenza spreads from urban to rural areas has been suggested by a few studies [30], [31] based on the observation that cases are detected in larger urban centers first. When the full epidemic wave is considered, urban and rural epidemics were found to be closely synchronized. These two views are not necessarily in disagreement, as, even with a slight over representation of the rural population, the community with the larger population will detect more cases, and hence be more likely to detect the first cases. Even if the epidemic starts with a larger per capita number of imported cases in the urban community, mixing between urban and rural areas should help force synchronization. In comparison to other studies where maps were produced with minimal data using spatial interpolation methods [12], [32], these maps for Canada and the United States illustrate that influenza activity is synchronized over large geographic areas with an occasional large delay between a few adjacent regions.

This study has several limitations. The lack of detailed geo-coding of laboratory confirmed influenza cases for some communities and the small number of confirmed cases for many cities limits the ability to clearly define influenza spread within populations and provide solid guidance on the geographic scale most appropriate for community level surveillance. As well, our analysis was based on only 6 seasons, not enough to fully assess the potential for variation in geographic spread. Patterns of geographic spread may also be different in mixed seasons with the circulation of multiple strains. While spread of influenza in rural and urban areas appeared to be synchronized, we were unable to assess the level of influenza activity in remote rural areas due to the very small number of people who live in remote rural communities. Cases in the case-based reporting database were identified by the date of specimen collection, while laboratory reports of influenza positive tests in the RVDSS were dated by the week of the test. Depending on laboratory procedures, a difference of a week or two was noted between the case-based reporting and the RVDSS. Finally, as specimens can be sent for testing for various reasons, we are uncertain about the temporal representativeness, though the general shape of the resulting epidemic curves [22] and the agreement between the weekly number of laboratory confirmations and excess hospital admissions [6] and deaths [33] suggest a reasonable temporal representativeness.

With the exception of some of the Atlantic Provinces in Canada, the number of confirmed cases per province per season was sufficient to accurately estimate the provincial timing of seasonal epidemic waves to within a week. Historic detection levels were also sufficient for the larger CMAs in most seasons, though higher testing rates would be needed to better assess the degree of geographic synchronization in surveillance areas with smaller populations. Considering the shape of the underlying epidemic curve and that 50% of the cases within a community occur within a 4–5 week period of peak activity [22], it should be feasible to detect the period of peak influenza activity in real time for finer surveillance areas, such as sub-provincial regions of the larger provinces in Canada or at state levels in the United States. As the available data was too sparse to fully describe the geographic areas for which local influenza activity could be expected to occur synchronously, dynamic transmission models might be able to incorporate the commuting flow data to advise on this issue.

In the six seasons analyzed, the epidemic peaked in at least one Canadian province before peaking in an American region in 3 out of 6 seasons analysed, though the epidemic waves spread across the United States in a shorter period of time than across Canada and a tendency for earlier onset in US regions compared to Canadian provinces was noted. At the community level, the anticipated delay in peak activity between large cities and rural areas was not observed. As social contact between regions appears to be too weak to predict the direction of spread and regional differences in timing, local surveillance, based on the proportion and number of positive tests, should form a solid basis for an early warning indicator of influenza activity in the community. Diffusion over large geographic areas appears to be the norm, though cannot be assumed. Despite these limitations, this analysis suggests that even at the community level, the weekly number of laboratory-confirmed cases can provide a very fine precision of the relative timing of influenza epidemics, and this data could be used to further enhance surveillance system reporting for urban and rural areas. While it is very likely that a few individual communities would experience unusually early or late epidemics compared to neighbouring major urban centres each season, it is worth noting that for smaller communities, on average, the best predictor of local activity in the current week could be the level of activity from the most recent weekly report for the region or neighbouring communities, rather than for the individual community. Hence the ideal spatial aggregation would have to be a balance between the purpose for which the surveillance data is to be used, the number and type of reports related to influenza activity for the proposed geographic unit and the likelihood of synchronization within the geographic unit. Activity in adjacent regions across international borders would be as relevant to this decision making as adjacent regions within national borders. A North American flu map or maps showing activity across contiguous areas would seem feasible and useful for these purposes.
